# Cardiac CaMKII*δ* and Wenxin Keli Prevents Ang II-Induced Cardiomyocyte Hypertrophy by Modulating CnA-NFATc4 and Inflammatory Signaling Pathways in H9c2 Cells

**DOI:** 10.1155/2020/9502651

**Published:** 2020-10-19

**Authors:** Na An, Yu Chen, Yanfen Xing, Honghua Wu, Xiongyi Gao, Hengwen Chen, Ke Song, Yuanyuan Li, Xinye Li, Fan Yang, Xiandu Pan, Xiaofang He, Xin Wang, Yang Li, Yonghong Gao, Yanwei Xing

**Affiliations:** ^1^Guang'anmen Hospital, Chinese Academy of Chinese Medical Sciences, Beijing 100053, China; ^2^Key Laboratory of Chinese Internal Medicine of Ministry of Education, Dongzhimen Hospital Affiliated to Beijing University of Chinese Medicine, Beijing 100700, China; ^3^Fujian Health College, Fuzhou 350101, China; ^4^Shanxi University of Chinese Medicine, Jinzhong 030619, China; ^5^Tianjin State Key Laboratory of Modern Chinese Medicine, Tianjin Key Laboratory of TCM Chemistry and Analysis Institute of Traditional Chinese Medicine, Tianjin University of Traditional Chinese Medicine, Tianjin 300193, China; ^6^Beijing University of Chinese Medicine, Beijing, China; ^7^Department of Cardiology, General Hospital of People's Liberation Army, Beijing 100853, China

## Abstract

Previous studies have demonstrated that calcium-/calmodulin-dependent protein kinase II (CaMKII) and calcineurin A-nuclear factor of activated T-cell (CnA-NFAT) signaling pathways play key roles in cardiac hypertrophy (CH). However, the interaction between CaMKII and CnA-NFAT signaling remains unclear. H9c2 cells were cultured and treated with angiotensin II (Ang II) with or without silenced CaMKII*δ* (siCaMKII) and cyclosporine A (CsA, a calcineurin inhibitor) and subsequently treated with Wenxin Keli (WXKL). Patch clamp recording was conducted to assess L-type Ca^2+^ current (I_Ca-L_), and the expression of proteins involved in signaling pathways was measured by western blotting. Myocardial cytoskeletal protein and nuclear translocation of target proteins were assessed by immunofluorescence. The results indicated that siCaMKII suppressed Ang II-induced CH, as evidenced by reduced cell surface area and I_Ca-L_. Notably, siCaMKII inhibited Ang II-induced activation of CnA and NFATc4 nuclear transfer. Inflammatory signaling was inhibited by siCaMKII and WXKL. Interestingly, CsA inhibited CnA-NFAT pathway expression but activated CaMKII signaling. In conclusion, siCaMKII may improve CH, possibly by blocking CnA-NFAT and MyD88 signaling, and WXKL has a similar effect. These data suggest that inhibiting CaMKII, but not CnA, may be a promising approach to attenuate CH and arrhythmia progression.

## 1. Introduction

Cardiomyocyte hypertrophy (CH) is an adaptive response to the pathological stimuli that maintain normal cardiac function. CH is a prerequisite marker of heart failure (HF) and usually occurs after myocardial infarction and stress overload. Although the initially adaptive response can maintain cardiac output, sustained hypertrophic growth can lead to a pathological state that leads to decreased compliance, HF, and sudden death [1–5]. Therefore, CH remains a major threat in the population, and it is necessary to elucidate the pivotal molecular mechanisms involved in CH that can ameliorate pathological CH responses. Different signaling molecules have been considered as causes of myocardial hypertrophy, including nuclear factor of activated T cells (NFAT), calcium-/calmodulin- (CaM-) dependent protein kinase II (CaMKII), and *β*-adrenergic receptors [6].

Calcineurin (CaN), a calcium- and CaM-dependent serine/threonine phosphatase, is a well-established mediator of *β*-adrenergic-induced CH [7, 8]. CaN consists of two subunits: the catalytic subunit (CnA) and a regulatory Ca^2+^-binding subunit (CnB) [9, 10]. It is generally known that CaN plays an influential role in regulating pathological hypertrophy. The constitutive activation of CaN and its downstream target, NFAT, are thought to play an important role in abnormal CH [11, 12]. Moreover, it is known that CaMKII phosphorylates many vital signaling factors that are related to initiating abnormal hypertrophy [13]. Transgenic overexpression of the splice variants CaMKII*δ*b (located in the nucleus) and CaMKII*δ*c (located in the cytosol) promotes CH and dilated cardiomyopathy, respectively. However, the complete pathological molecular mechanism is not fully understood, which hinders the development of improved treatments for CH [14, 15]. Ca^2+^-dependent signaling through CaMKII and CaN has been suggested to contribute to adverse CH [16]. However, the interaction between CaMKII and CnA-NFAT signaling for CH remains unclear.

**Figure 1:**
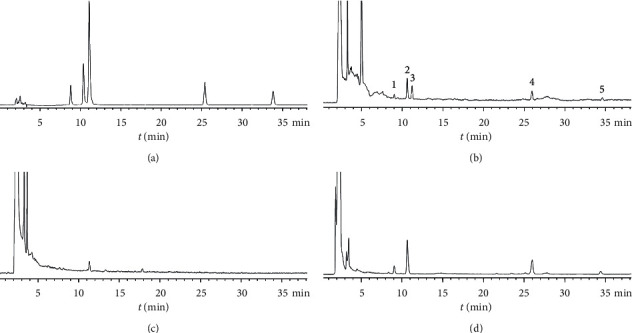
The HPLC chromatograms of WXKL. (a) Chemical reference substances, (b) WXKL, (c) negative samples of Panax notoginseng, and (d) *Codouopsis pilosula*. (1) notoginsenoside R1; (2) ginsenoside Rg1; (3) obetyolin; (4) ginsenoside Rb1; (5) ginsenoside Rd.

Wenxin Keli (WXKL) is the first Chinese medicine approved by the Chinese state for its antiarrhythmic effects; the medicine can tonify *q*_*i*_, supply *y*_in_, promote blood circulation, and remove blood stasis according to the theory of traditional Chinese medicine. The main components of WXKL consist of *Nardostachys jatamansi (D*.*Don) DC* (Gansong), *Codonopsis pilosula (Franch*.*) Nannf* (Dangshen), *Panax notoginseng (Burkill) F*.*H*.*Chen* (Sanqi), *Succinum* (Hupo), and *Polygonatum cyrtonema Hua* (Huangjing). According to the national pharmacopoeia [17], WXKL mainly contains notoginseng saponin R1 (C_47_H_80_O_18_), ginseng saponin Rg1 (C_42_H_72_O_14_), and ginseng saponin Rb1 (C_54_H_92_O_23_).  Figure 1[18] displays the HPLC chromatograms of the chemical reference substances, WXKL, and negative samples of *Panax notoginseng* and *Codonopsis pilosula*. Previous studies have shown that WXKL may inhibit HF and arrhythmia by regulating the CaMKII signaling pathway [19–23]. WXKL in the treatment of patients with HF or arrhythmia can improve the exercise tolerance and is beneficial for the recovery of cardiac function [24–26]. However, to the best of our knowledge, whether WXKL improves CH via regulating the CaMKII and CnA-NFAT signaling pathways has not been investigated.

Given the pivotal roles of the CaMKII and CaN signaling pathways in the regulation of abnormal hypertrophy, we selected H9c2 cells induced by angiotensin II (Ang II) and used CaMKII silencing and treatment with cyclosporine A (CsA, a CaN inhibitor) to explore the correlation between the CaMKII and CnA-NFAT signaling pathways. Additionally, we sought to determine whether WXKL could improve HF by regulating CaMKII and CnA-NFAT.

## 2. Materials and Methods

### 2.1. Construction of Silenced CaMKII*δ* in H9c2-1632 Cells

H9c2 rat embryonic cardiomyocyte cells were obtained from the Institute of Basic Medical Sciences, Chinese Academy of Medical Sciences (Cell Resource Center, IBMS, CAMS/PUMC, China). First, RNA interference at the site of the target gene, CaMKII*δ* (Rat), was performed. According to the target gene sequence site, four sequences with the target gene were inserted into the vector pCDNA6.2 emGFP, and miRNA reverse primer was used as the sequencing primer (Table 1). Sequencing results verified that the correct sequence was inserted. Transfection and preparation of plasmids, blasticidin screening, and determination of the concentration of H9c2 cells, and finally cell transfection and screening were performed. Real-time PCR results showed that the relative mRNA expression of H9c2-1141 and H9c2-1632 sites was significantly reduced after RNA interference (Figure S2A), and the inhibition rate of relative mRNA expression after H9c2-1632 site interference was slightly lower than that of H9c2-1141, but the difference was not statistically significant (Table 2). CaMKII*δ* protein expression was determined by western blot analysis, and the results showed that, after performing RNA interference at four sites, CaMKII*δ* protein expression was decreased, with the greatest decrease at the H9c2-1632 site (Figure S2B). Combined with real-time PCR, the H9c2-1632 site was finally selected as a target to construct a CaMKII*δ* (Rat) RNA interference cell line. The H9c2-1632 cells were cultured in an incubator containing 5% CO_2_ in high-sugar Dulbecco's modified Eagle's medium containing 10% fetal bovine serum at 37°C.

### 2.2. Drugs and Solutions

Ang II (A9525, Sigma Co., St. Louis, MO, USA) was dissolved in deionized water at a concentration of 10^−5^ mol/L; 20 *μ*L of this solution was added to the culture medium (2 mL), reaching a final concentration of 10^−7^ mol/L. CsA (Sigma Co.) was dissolved in ethyl alcohol at a concentration of 10^−4^ mol/L; 20 *μ*L of this solution was added to the culture medium (2 mL) at a final concentration of 10^−6^ mol/L. WXKL (1910051, Shandong Buchang Pharmaceuticals Co., Ltd., Shandong, China) was dissolved in saline at a concentration of 5 g/L.

In HPLC, 1 g of finely ground WXKL was added to a conical flask with a stopper, 50 ml of water-saturated *n*-butanol was added, and the flask was tightly stoppered and weighed. After soaking for 12 hours and ultrasonic treatment (power 300 W, frequency 40 kHz) for 1 h, the flask was weighed again after cooling, water-saturated *n*-butanol was added to make up for the lost weight, and the flask was shaken well. After filtration, 25 ml of the filtrate was collected. The *n*-butanol solution was evaporated to dryness, and methanol solution was added to the residue to 10 ml and shaken. According to the proportion of prescription Chinese medicine, *notoginseng* and *Codonopsis* were removed separately to make negative samples of *Panax notoginseng* and *Codouopsis pilosula*, and the negative control solution was prepared according to the previously mentioned methods. Methanol was added to prepare reference substances containing notoginsenoside R1 0.247 mg, ginsenoside Rg1 0.422 mg, ginsenoside 0.042 mg, ginsenoside Rb1 0.847 mg, and ginsenoside Rd 0.255 mg per 1 mL.

### 2.3. Chromatographic Conditions

Analyses were performed on an Agilent 1260 HPLC system consisting of a quaternary delivery system, an autosampler, and a DAD detector. All the separations were carried out on Amethyst C_18_ column (4.6 × 250 mm, 4 *μ*m). The gradient elution used acetonitrile (A) and water (B) as a mobile phase at a flow rate of 1 mL/min. The gradient program was as follows: 0–14 min, 22%–30%A; 14–35 min, 30%–38%A; 35–45 min, 38%–38%A; 45–47 min, 38%–95%A; 47–62 min, 95%–95%A; 62–65 min, 95%–22%A. The column temperature was maintained at 27°C, and the chromatogram was monitored at a wavelength of 210 nm.

### 2.4. Cell Grouping and Drug Administration

H9c2 rat embryonic cardiomyocytes were split into 13 different treatment groups: (1) negative control group: only secondary antibody was added to H9c2 cells with no primary antibody; (2) control group: H9c2 cells were cultured for 72 h; (3) control + Ang II group: H9c2 cells were pretreated with Ang II for 48 h and cultured for another 24 h; (4) control + Ang II + WXKL group: H9c2 cells were pretreated with Ang II for 48 h, treated with WXKL, and cultured for another 24 h; (5) control + WXKL group: H9c2 cells were cultured for 48 h, treated with WXKL, and cultured for another 24 h; (6) siCaMKII group: H9c2-1632 cells were cultured for 72 h; (7)siCaMKII + Ang II group: H9c2-1632 cells were pretreated with Ang II for 48 h and cultured for another 24 h; (8) siCaMKII + Ang II + WXKL group: H9c2-1632 cells were pretreated with Ang II for 48 h, treated with WXKL, and cultured for another 24 h; (9)siCaMKII + WXKL group: H9c2-1632 cells were cultured for 48 h, treated with WXKL, and cultured for another 24 h; (10) CsA group: H9c2 cells were pretreated with CsA for 72 h; (11) CsA + Ang II group: H9c2 cells were pretreated with CsA and Ang II for 48 h and cultured for another 24 h; (12) CsA + Ang II + WXKL group: H9c2 cells were pretreated with CsA and Ang II for 48 h, treated with WXKL, and cultured for another 24 h; (13) CsA + WXKL group: H9c2 cells were pretreated with CsA for 48 h, treated with WXKL, and cultured for another 24 h.

### 2.5. Western Blot Analysis

Proteins were extracted from H9c2 cells, subsequently lysed with lysis buffer containing phenylmethylsulfonyl fluoride in ice for 40 min, and shaken once every 8 min during this period. The lysates were centrifuged at 1200 rpm for 20 min at 4°C, and the supernatants were collected. Proteins were analyzed by sodium dodecyl sulfate-polyacrylamide gel electrophoresis, transferred onto nitrocellulose membranes, blocked in 5% bovine serum albumin (BSA) or milk, and then incubated with primary antibodies at 4°C overnight. Subsequently, the membranes were washed three times with tris-buffered saline/Tween 20 at the specified time intervals and finally incubated with secondary antibody at room temperature. ECL visualization was performed, and the resulting images were captured using the GeneGnome Gel Imaging System (Syngene Co., Bangalore, India). The gel images were analyzed using ImageJ software (Image-Pro Plus, Media Cybernetics, Rockville, MD, USA). The antibodies used in the present study are listed in Table S1.

### 2.6. Confocal Imaging

For the cytoskeletal assay, H9c2 cells were cultured in a confocal laser culture dish until reaching a moderate density. Upon reaching 70–80% confluence, the cells were washed with phosphate-buffered saline (PBS) twice, fixed with 4% paraformaldehyde for 15 min, and washed with PBS three times for 5 min each. Cells were incubated with 0.1% Triton X-100 penetrating fluid at room temperature for 5 min and washed thrice with PBS for 5 min each. Rhodamine-phalloidin was diluted with PBS (2.5 *μ*L) and added to 200 *μ*L of the working fluid, fixed for 20 min, and washed with PBS three times for 5 min each. Cells were subjected to confocal laser microscopy. The cells of each group were removed and washed with cold PBS (precooled at 4°C) twice, and the residual PBS was aspirated. Each group was fixed with 4% paraformaldehyde for 10 min at −20°C and washed twice with PBS. Cells were permeabilized with 0.25% Triton X-100 for 10 min and washed three times with cold PBS for 5 min each wash and then treated with 1% BSA for 30 min to block nonspecific antigen recognition. Primary antibodies were diluted with 1% BSA in PBS and added at 100 *μ*L to each group. Cells were plated at 4°C for 12 h and then washed thrice with PBS for 5 min each. The secondary antibody was diluted with 1% BSA in PBS with 100 *μ*L per sample at room temperature for 60 min away from light. The secondary antibody was removed and cells were washed thrice with PBS for 5 min each. Cells were incubated with DAPI for nuclear staining; finally, an antifluorescence quencher was added. Later, a transparent nail polish seal was used, and cells were observed using a confocal microscope.

For measuring the nuclear translocation of NFATc4, the cell processing method is basically the same as previously mentioned. Images were analyzed using Image-Pro Plus Analysis Software. First, color images were converted to gray images. The ratio of IOD to the area represented by NFATc4 fluorescence was measured. NFATc4 translocation was quantified as the ratio between the number of cells containing nucleus-localized NFATc4 (NFATc4-positive), and the total number of cells was counted.

### 2.7. Electrophysiological Recording

Using the whole-cell patch clamp technique, the whole-cell Ca^2+^ current was recorded by an Axon-700B amplifier (Axon Instruments, San Jose, CA, USA) with the pCLAMP 9.2 software (Axon Instruments). Borosilicate glass microelectrodes had tip resistances of 3.0–5.0 MΩ, which adjusts the three-dimensional manipulator for GΩ sealing and breaks the membrane absorption in the whole-cell recording mode. The membrane capacitance and I_Ca-L_ current were recorded after stabilization. To eliminate the errors resulting from cell size, the I value was expressed as the current density (pA/pF). Cells were superfused with extracellular fluid containing (all in mmol/L): 125 NaCl, 10.8 BaCl_2_, 1 MgCl_2_, 5.4 CsCl, 10 HEPES, and 10 glucose (pH 7.35, adjusted with NaOH). A pipette solution was used containing (mmol/L): 120 CsCl, 3 MgCl_2_, 5 Na_2_ATP, 10 EGTA, and 5 HEPES (pH 7.3, adjusted with CsOH).

### 2.8. Statistical Analysis

Data were expressed as mean ± SD. One-way ANOVA was used to compare multiple groups with a normal distribution. Statistical analysis was performed using the SPSS program (version 20.0). *P* < 0.05 was considered statistically significant, and *P* < 0.01 was considered highly statistically significant. Data acquisition and analysis were performed using pCLAMP 9.2 software (Axon Instruments), Origin 6.1 software (MicroCal Software, Northampton, MA, USA), and GraphPad Prism 5 (GraphPad Software Incorporate, La Jolla, CA, USA).

## 3. Results

We explored the interaction between CaMKII and CnA-NFAT signaling in CH induced by Ang II and the effect of WXKL. Therefore, we sought to characterize the role of CaMKII and CnA-NFAT signaling in cardiomyocytes using siRNA-mediated silencing (siCaMKII) and CsA. We analyzed the results of cytoskeletal enlargement; L-type Ca^2+^ current (I_Ca-L_); expression of CaMKII, CnA-NFAT, and inflammatory signaling pathways in cardiomyocytes induced by Ang II; and the protein expression and nuclear transfer of NFATc4.

### 3.1. Effects of SiCaMKII and CsA on Myocardial Cytoskeletal Protein

We used fluorescence confocal microscopy to observe cytoskeletal enlargement (a direct indicator of hypertrophy in myocardial cells) to assess whether the CH model had been successfully established and ensure the feasibility of the experiment.

**Figure 2:**
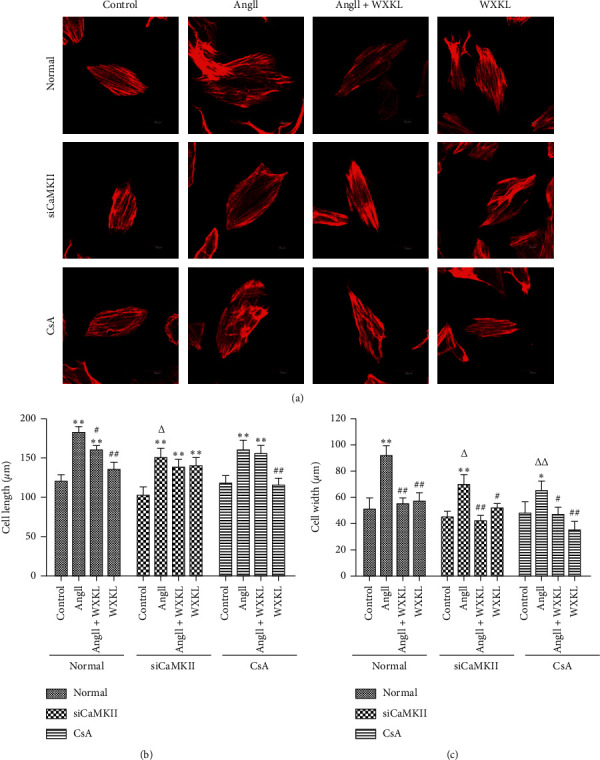
SiCaMKII decreases Ang II-induced cell surface area enlargement in H9c2 cells. H9c2 cells were treated with Ang II (10^−7^ M for 48 h). CaMKII was silenced or CsA (10^−6^ M) was added to the culture medium prior to Ang II administration. After administering Ang II, WXKL (5 g/L) was added, and the culture medium was incubated for 24 h. The control cells received no treatment. (a) Representative images of immunofluorescence staining for phalloidin following treatment (*n* = 10 cells per group). Scale bar: 30 *μ*m. (b) Mean cell length measurement (*n* = 10 cells per group). (c) Mean cell width measurement (*n* = 10 cells per group). Data are presented as the mean ± SD. Statistical significance was determined by one-way ANOVA. ^*∗*^*P* < 0.05 and ^*∗∗*^*P* < 0.01 vs. the control group. ^#^*P* < 0.05 and ^##^*P* < 0.01 vs. the Ang II group. ^▲^*P* < 0.05 and ^▲▲^*P* < 0.01; the control group in SiCaMKII or CsA vs. the control group in normal. ^△^*P* < 0.05 and ^△△^*P* < 0.01; the Ang II group in SiCaMKII or CsA vs. the Ang II group in normal.

Immunofluorescence staining showed that Ang II effectively induced CH, as evidenced by an increase in cell width and length (Figure 2). CsA and siCaMKII prevented an Ang II-stimulated increase in cell size, and the effect was similar in cells treated with WXKL (Figure 2). Taken together, the results revealed that siCaMKII and CsA may inhibit the hypertrophic response to Ang II in H9c2 cells. Moreover, WXKL improved Ang II-induced CH.

### 3.2. I_Ca-L_ Significantly Reduced after CaMKII*δ* Silencing

I_Ca-L_ is present in many cardiomyocytes, and it plays a key role in the formation of an action potential plateau, intracellular Ca^2+^ elevation, and muscle contraction. CaM, as a Ca^2+^ receptor, plays a major role in Ca^2+^-dependent inactivation and facilitation of I_Ca-L_.

**Figure 3:**
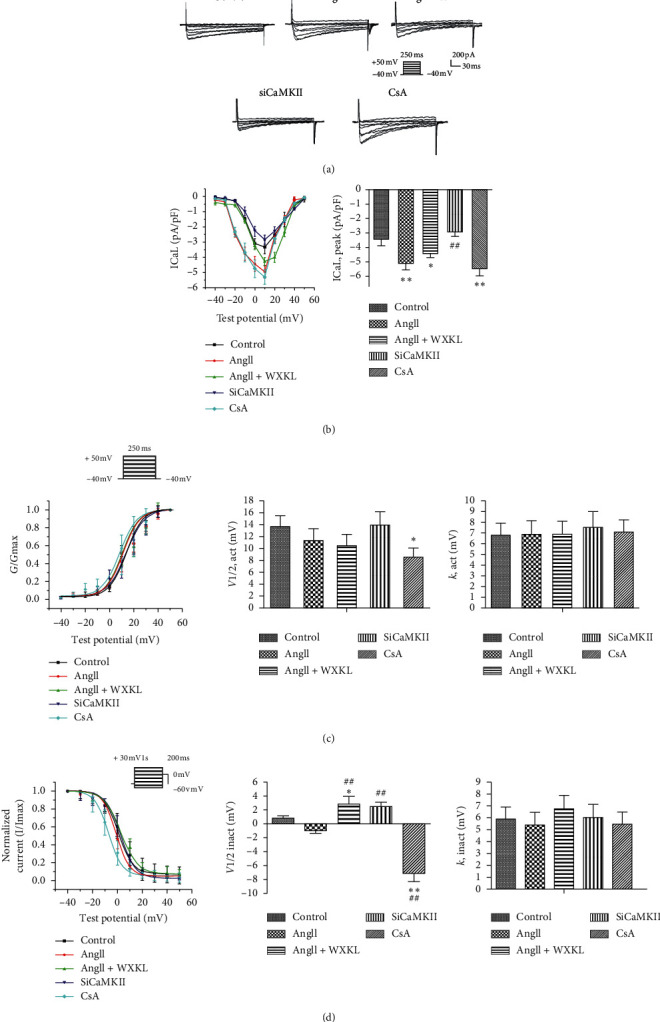
Changes in patch clamp inspection. (a) I_Ca-L_ current in the control, Ang II, Ang II + WXKL, SiCaMKII, and CsA groups. (b) Effects of siCaMKII and CsA on the I_Ca-L_ current-voltage (I–V) curve in H9c2 cells. I–V curves and peak current density (PA/PF) of each group (*n* = 10 cells per group). (c) Effects of siCaMKII and CsA on I_Ca-L_ steady-state activation (SSA) in H9c2 cells. I_Ca-L_ steady-state activation curves (original data point diagram and curve fit by Boltzmann equation), comparison of semiactivation voltage (*V*_1/2, act_), and comparison of slope factors (K_,act_) in each group (*n* = 10 cells per group). (d) Effects of siCaMKII and CsA on I_Ca-L_ steady-state inactivation in H9c2 cells. I_Ca-L_ steady-state inactivation curves (original data point diagram and curve fitting by Boltzmann equation); comparison of semi-inactivation voltage (*V*_1/2, inact_); comparison of slope factors (K_,inact_) in each group (*n* = 10 cells per group). Values are presented as mean ± SD; Statistical significance was determined by one-way ANOVA. ^*∗*^*P* < 0.05 and ^*∗∗*^*P* < 0.01 vs. the control group. ^#^*P* < 0.05 and ^##^*P* < 0.01 vs. the Ang II group.

Results revealed that compared with control treatment, Ang II significantly increased the amplitude of I_Ca-L_ within the range of −20 mV to +10 mV. Pretreatment with siCaMKII reduced the elevated I_Ca-L_ amplitude. Similarly, WXKL decreased the Ang II-induced elevated amplitude of I_Ca-L_ (Figures 3(a) and 3(b)). Interestingly, in the CsA group (Figure 3), I_Ca-L_ was significantly increased, and its steady-state activation and inactivation were increased (Figures 3(c) and 3(d)). These results suggest that siCaMKII and WXKL inhibit the calcium current and impede inactivation. Furthermore, treatment with CsA led to the opposite results. Therefore, we suspected that CaN inhibition may alter other pathways and cause an increase in calcium current.

### 3.3. Treatment with CsA Activates the CaMKII Signaling Pathway

To further investigate Ang II-induced CaMKII expression in H9c2 cells and the effect of drug intervention on CaMKII protein expression, we measured CaMKII protein levels using laser scanning confocal microscopy. Additionally, CaMKII signaling pathway proteins were subjected to western blotting to explore the mechanism.

**Figure 4:**
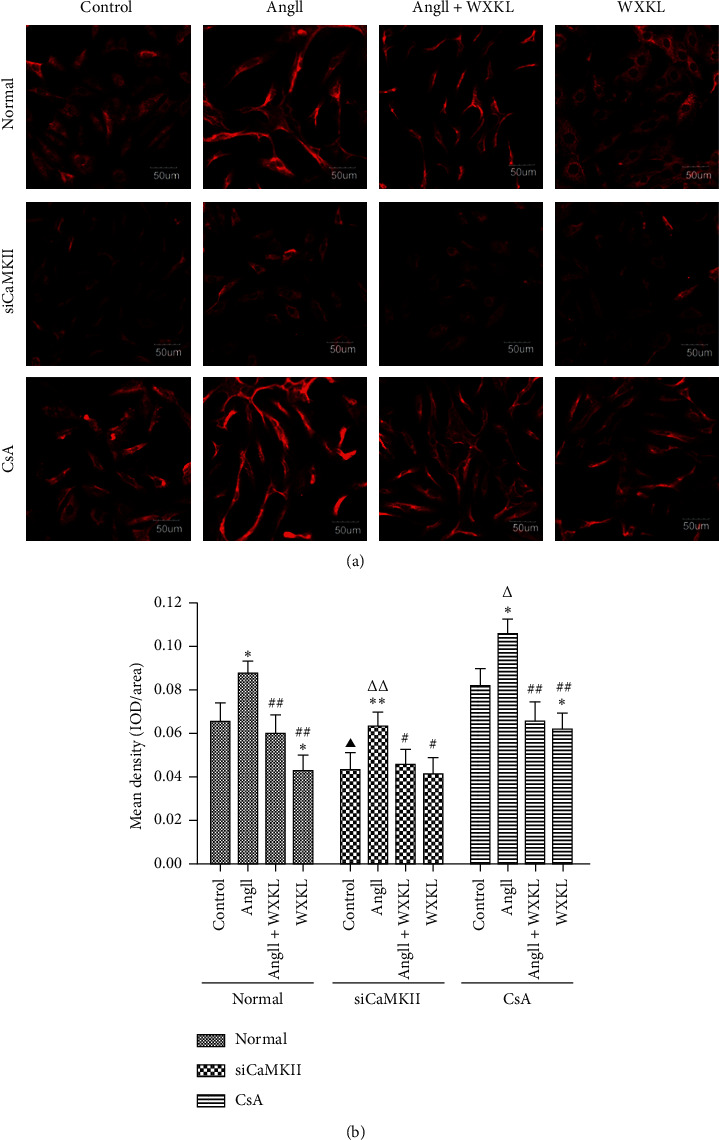
Effects of siCaMKII and CsA on the CaMKII signaling cascade. (a) Double immunofluorescence staining to observe the effects of siCaMKII, CsA, and WXKL on CaMKII expression in each group. Scale bar: 50 *μ*m. (b) Mean density of CaMKII in each group. Data are presented as the mean ± SD. Statistical significance was determined by one-way ANOVA. ^*∗*^*P* < 0.05 and ^*∗∗*^*P* < 0.01 vs. the control group. ^#^*P* < 0.05 and ^##^*P* < 0.01 vs. the Ang II group. ^▲^*P* < 0.05 and ^▲▲^*P* < 0.01; the control group in SiCaMKII or CsA vs. the control group in normal. ^△^*P* < 0.05 and ^△△^*P* < 0.01; the Ang II group in SiCaMKII or CsA vs. the Ang II group in normal.

The results revealed that Ang II treatment significantly increased the fluorescence intensity of CaMKII in H9c2 cells compared with control treatment (Figure 4(a)). Pretreatment with siCaMKII reduced the Ang II-induced elevation in the fluorescence intensity of CaMKII, and WXKL led to a similar decrease. However, the fluorescence intensity corresponding to the CaMKII protein level was significantly higher in the CsA group than in the normal group (Figure 4(b)) and was observed to decrease after WXKL treatment. Therefore, the results showed that the fluorescence intensity indicating CaMKII protein expression increased after stimulation with Ang II, whereas WXKL treatment significantly reduced this increase. However, treatment with CsA did not inhibit this effect.

**Figure 5:**
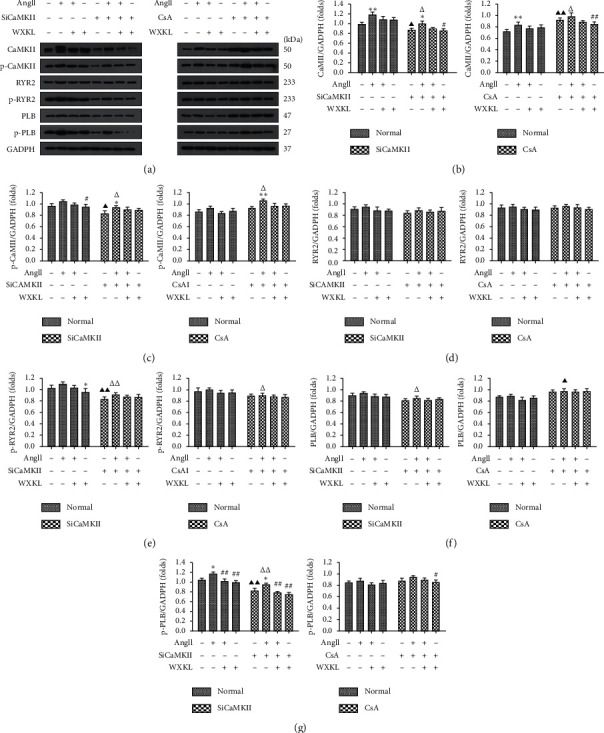
Effects of siCaMKII and CsA on Ang II-induced activation of CaMKII signaling in H9c2 cells. (a) Representative western blotting images. Densitometric analysis of (b) CaMKII, (c) p-CaMKII, (d) RyR2, (e) p-RyR2, (f) PLB, and (g) p-PLB expression levels (*n* = 3 cells per group). Data are presented as the mean ± SD. Statistical significance was determined by one-way ANOVA. ^*∗*^*P* < 0.05 and ^*∗∗*^*P* < 0.01 vs. the control group. ^#^*P* < 0.05 and ^##^*P* < 0.01 vs. the Ang II group. ^▲^*P* < 0.05 and ^▲▲^*P* < 0.01; the control group in SiCaMKII or CsA vs. the control group in normal. ^△^*P* < 0.05 and ^△△^*P* < 0.01; the Ang II group in SiCaMKII or CsA vs. the Ang II group in normal.

The expression of CaMKII, p-CaMKII, RyR2, p-RyR2, PLB, and p-PLB in H9c2 cells after stimulation with Ang II for 48 h was evaluated using western blot analysis. Exposure of H9c2 cells to Ang II resulted in increased expression of CaMKII pathway proteins (Figure 5(a)). Pretreatment with siCaMKII significantly decreased the Ang II-induced elevated expression of p-RyR2 and p-PLB (*P* < 0.01, Figures 5(e) and 5(f)) and reduced that of CaMKII and p-CaMKII (*P* < 0.05, Figures 5(b) and 5(c)). Interestingly, pretreatment with CsA led to significantly upregulated CaMKII expression levels (*P* < 0.01, Figure 5(b)). After treatment with WXKL for 24 h, protein expression was decreased in each group. Therefore, the CaMKII signaling pathway was blocked by silencing CaMKII expression, which reduced the degree of hypertrophy, and WXKL had a similar effect. However, the CaMKII signaling pathway was activated in H9c2 cells treated with CsA.

### 3.4. CaMKII Controls the CnA-NFAT Pathway

Next, we elucidated the effects of siCaMKII, CsA, and WXKL on the CnA-NFAT signaling pathway.

Treatment with Ang II significantly increased the fluorescence intensity of CnA in both the nucleus and the cytoplasm (Figure S1). The siCaMKII cells showed downregulation of CnA protein levels and lower fluorescence intensities. After treatment with WXKL for 24 h, Ang II-induced CnA protein expression was inhibited, and its fluorescence intensity and nuclear transfer were reduced (Figure S1). These results revealed that the fluorescence intensity and nuclear transfer of CnA in the Ang II-treated group were significantly higher; however, CaMKII silencing as well as treatment with CsA and WXKL suppressed this effect.

**Figure 6:**
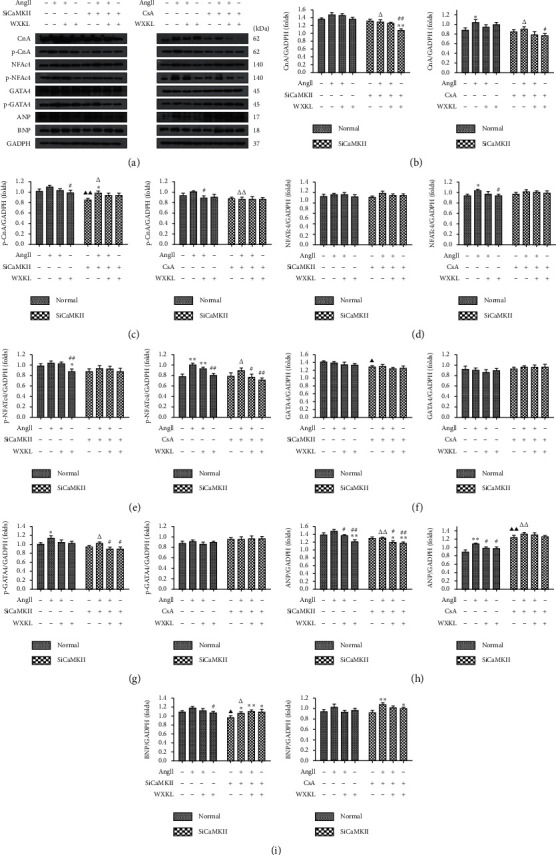
Effects of siCaMKII and CsA on Ang II-induced activation of CnA-NAFT signaling in H9c2 cells. (a) Representative western blotting images. Densitometric analysis of (b) CnA, (c) p-CnA, (d) NFATc4, (e) p-NFATc4, (f) GATA4, (g) p-GATA4, (h) ANP, and (i) BNP expression levels (*n* = 3 cells per group). Data are presented as the mean ± SD. Statistical significance was determined by one-way ANOVA. ^*∗*^*P* < 0.05 and ^*∗∗*^*P* < 0.01 vs. the control group. ^#^*P* < 0.05 and ^##^*P* < 0.01 vs. the Ang II group. ^▲^*P* < 0.05 and ^▲▲^*P* < 0.01; the control group in SiCaMKII or CsA vs. the control group in normal. ^△^*P* < 0.05 and ^△△^*P* < 0.01; the Ang II group in SiCaMKII or CsA vs. the Ang II group in normal.

As shown in Figure 6(a), the expression of upstream and downstream proteins, including CnA, p-CnA, NFATc4, p-NFATc4, GATA4, p-GATA4, ANP, and BNP, in the CnA-NFAT signaling pathway was detected by western blotting. Exposure of H9c2 cells to Ang II resulted in increased expression of CnA-NFAT signaling pathway proteins (Figure 6). Pretreatment with siCaMKII markedly decreased the Ang II-induced elevated expression of p-CnA (*P* < 0.01, Figure 6(c)) and reduced that of GATA4 and BNP (*P* < 0.05, Figures 6(f) and 6(i)). Interestingly, pretreatment with CsA led to significantly upregulated ANP expression levels (*P* < 0.01, Figure 6(h)). After treatment with WXKL for 24 h, protein expression was decreased in each group. Therefore, the CnA-NFAT signaling pathway was blocked by silencing CaMKII expression, and WXKL played a role in improving the expression of various proteins in CH.

### 3.5. SiCaMKII Inhibits Nuclear Transfer of NFATc4 in Hypertrophic Cardiomyocytes

Subsequently, we further analyzed CnA-NFAT signaling, and immunofluorescence and western blots were performed to detect NFATc4 nuclear translocation.

**Figure 7:**
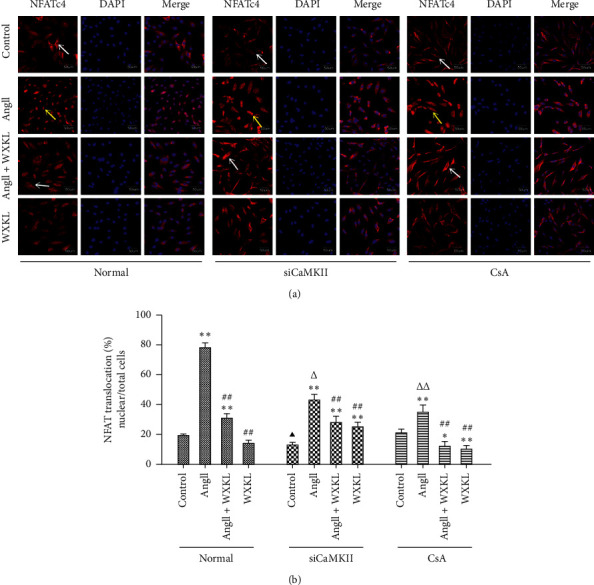
Ang II-induced NFATc4 nuclear translocation is inhibited by siCaMKII. H9c2 cells were treated with Ang II (10^−7^ M) for 48 h. CaMKII was silenced or CsA (10^−6^ M) was added to the culture medium prior to Ang II administration. WXKL (5 g/L) was added for 24 h after Ang II administration. The control cells received no treatment. (a) Representative images of immunofluorescence staining of NFATc4. Blue: DAPI staining; red: NFATc4. Scale bar: 50 *μ*m. Yellow arrow: nuclear translocation; white arrow: no nuclear translocation or nuclear translocation was decreased. (b) Semiquantitative analysis of NFATc4 nuclear translocation. Data are presented as the mean ± SD. Statistical significance was determined by one-way ANOVA. ^*∗*^*P* < 0.05 and ^*∗∗*^*P* < 0.01 vs. the control group. ^#^*P* < 0.05 and ^##^*P* < 0.01 vs. the Ang II group. ^▲^*P* < 0.05 and ^▲▲^*P* < 0.01; the control group in SiCaMKII or CsA vs. the control group in normal. ^△^*P* < 0.05 and ^△△^*P* < 0.01; the Ang II group in SiCaMKII or CsA vs. the Ang II group in normal.

The results demonstrated that NFATc4 was translocated to the nucleus in response to Ang II stimulation (Figure 7), whereas pretreatment with siCaMKII or CsA inhibited Ang II-induced nuclear translocation of NFATc4; furthermore, such transfer was inhibited after treatment with WXKL. The data presented in Figures 6 and 7 suggest that inhibition of hypertrophy by siCaMKII and WXKL was mediated, at least in part, by CaMKII and CnA-NFATc4 signaling.

### 3.6. Effects of SiCaMKII and CsA on the Inflammatory Signaling Pathway (MyD88-TLR4)

**Figure 8:**
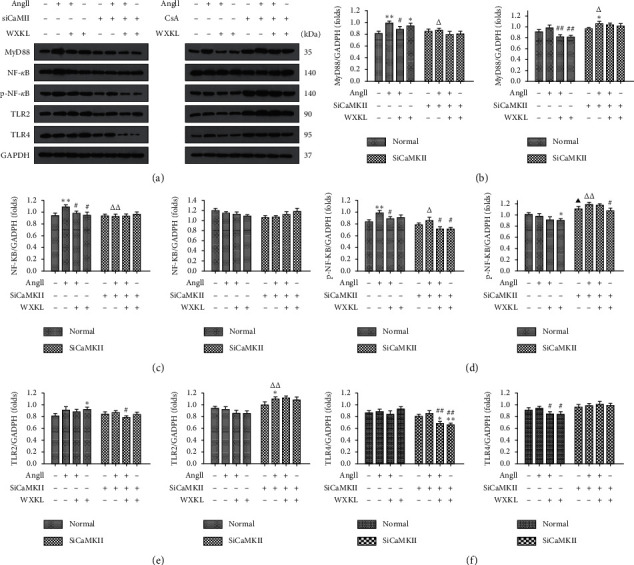
Effects of siCaMKII and CsA on Ang II-induced activation of the inflammatory signal transduction pathway in H9c2 cells. (a) Representative western blotting images. Densitometric analysis of (b) MyD88, (c) NF-*κ*B, (d) p-NF-*κ*B, (e) TLR2, and (f) TLR4 expression levels (*n* = 3 cells per group). Data are presented as the mean ± SD. Statistical significance was determined by one-way ANOVA. ^*∗*^*P* < 0.05 and ^*∗∗*^*P* < 0.01 vs. the control group. ^#^*P* < 0.05 and ^##^*P* < 0.01 vs. the Ang II group. ^▲^*P* < 0.05 and ^▲▲^*P* < 0.01; the control group in SiCaMKII or CsA vs. the control group in normal. ^△^*P* < 0.05 and ^△△^*P* < 0.01; the Ang II group in SiCaMKII or CsA vs. the Ang II group in normal.

Finally, to further elucidate the interaction between the CaMKII and CnA-NFAT signaling pathways, western blotting was used to measure the expression of MyD88, NF-*κ*B, p-NF-*κ*B, TLR2, and TLR4 in different groups (Figure 8).

The results demonstrated that protein expression was increased in H9c2 cells after Ang II treatment for 48 h. A statistically significant difference was observed in the expression of MyD88, NF-*κ*B, and p-NF-*κ*B (*P* < 0.01, Figures 8(b), 8(c), and 8(d)). Compared with that after Ang II treatment in normal H9c2 group, inflammation-related protein expression decreased after that in the siCaMKII group. In the CsA group, NF-*κ*B expression was slightly decreased whereas that of other proteins was increased. After treatment with Ang II for 48 h, the rate of increase of MyD88 and TLR2 was higher in the CsA group than that in H9c2 cells. After administering WXKL, protein expression decreased in each group. The previously mentioned findings indicated that, in the siCaMKII group, protein expression reduced in Ang II-induced cardiomyocytes and the inflammatory pathway was inhibited, but the opposite result was observed in the CsA group. Furthermore, the effect of WXKL was similar to that of siCaMKII.

## 4. Discussion

In the present study, we found that [1] Ang II activated the CaMKII, CnA-NFAT, and MyD88 inflammatory pathways in H9c2 cells and caused myocardial hypertrophy [2]. The siRNA-mediated silencing of CaMKII inhibited protein expression in the CaMKII pathway, further attenuated protein expression in the CnA-NFAT signaling pathway, and inhibited the reduction in NFATc4 nuclear transfer. Moreover, CaMKII silencing played a role in improving Ang II-induced myocardial hypertrophy, and WXKL had a similar effect [3]. CsA, a CaN inhibitor, inhibited expression in the CnA-NFAT pathway but activated the CaMKII and MyD88 signaling pathways.

**Figure 9:**
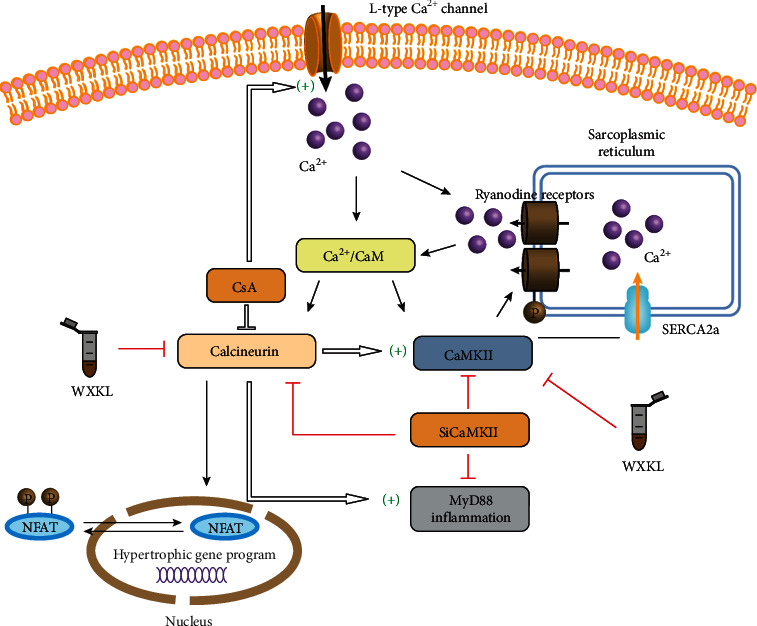
Ca^2+^-dependent signaling pathways in cardiac hypertrophy. Upon activation by CaM, CaN dephosphorylates cytoplasmic NFAT, a known hypertrophic transcription factor, and promotes its translocation to the nucleus and subsequent transcriptional activity. After silencing CaMKII, protein expression in the CaMKII and CaN-NFAT signaling pathways was inhibited, and the nuclear transfer of NFATc4 was decreased. CsA, an inhibitor of CaN, inhibits the expression in the CaN-NFAT pathway but activates the CaMKII signaling pathway, MyD88 inflammatory pathway, and I_Ca-L_. WXKL improves CH through the CaMKII and CaN-NFAT signaling pathways. CaM: calmodulin; CaN: calcineurin; NFAT: nuclear factor of activated T cells; CaMKII: Ca^2+^/calmodulin-dependent protein kinase II; L-type Ca^2+^ current: I_Ca-L_; CsA: cyclosporine A; WXKL: Wenxin Keli; CH: cardiac hypertrophy.

Both physiological and pathological CH are related to the increase in cardiomyocyte Ca^2+^ levels. Ca^2+^ regulates the activity of various Ca^2+^-dependent signaling pathways, including those of CaMKII and CnA-NFAT [6] (Figure 9). It is known that CaMKII phosphorylates class II histone deacetylases (HDACs), particularly HDAC4 and HDAC5, by boosting the export of these molecules from the nucleus, resulting in the disinhibition of MEF-2-mediated gene expression and CH [27–30]. CnA can maintain NFAT activity through a noncatalytic mechanism by associating with NFAT, blocking its nuclear export sequence and thereby maintaining its nuclear localization [31]. Once activated by sustained elevation of intracellular calcium, CaN dephosphorylates NFAT, enabling its translocation to the nucleus and consequently activating prohypertrophic target genes [32]. Our data showed that Ang II stimulation results in NFATc4 translocated to the nucleus, whereas pretreatment with siCaMKII or CsA inhibited Ang II-induced nuclear translocation of NFATc4; furthermore, transfer was inhibited after treatment with WXKL.

Previous research has shown that CaMKII inhibition can decrease Ang II-induced cardiac fibroblast proliferation and the secretion of TGF-*β*1 and TNF-*α*. In addition, CaMKII inhibition reversed the upregulation of MMP-1, 2, and 9 and collagen I and III after Ang II intervention [33]. A previous study revealed that treatment with KN-93 (a CaMKII inhibitor) significantly reduced the expression of CH-related proteins, including NFATc3, p-HDAC4, p-HDAC5, GATA-4, and the hypertrophy marker BNP. Furthermore, the combined inhibition of the CaMKII and CaN signaling pathways may obviously relieve CH responses [13]. The results of the current study revealed that siCaMKII inhibited protein expression in the CaMKII pathway and further reduced that in the CnA-NFAT signal pathway, thereby improving Ang II-induced hypertrophic cardiomyocytes. This was similar to the results of previous studies that demonstrated that, in the absence of CaMKII signals, CaN does not seem to contribute to abnormal cardiac remodeling, thus highlighting CaMKII and not CaN as a promising drug target to combat HF [16].

Excess CaMKII activity leads to phosphorylation of the L-type Ca^2+^ channel (LTCC), sarcoplasmic-endoplasmic reticulum Ca^2+^-ATPase, ryanodine receptor 2 (RyR2), and PLB proteins. CaMKII phosphorylates the LTCC that leads to increased I_Ca-L_ and forms a Ca^2+^ overload in the cell, which causes early afterdepolarizations and triggers arrhythmia. Hyperphosphorylation of the sarcoplasmic reticulum (SR) results in the consumption of SR Ca^2+^ stores, leading to damaged cytosolic Ca^2+^ transients, which in turn induces systolic and diastolic dysfunction. Moreover, hyperphosphorylation events at RyR2 cause abnormal release of SR Ca^2+^ that activates the electrogenic Na^+^/Ca^2+^-exchanger (NCX), which can cause delayed afterdepolarizations [34]. Interestingly, our study revealed that CnA expression was gradually reduced after treatment with CsA, but the CaMKII signaling pathway tended to be activated. This result was consistent with few studies that found either no effect or a deterioration of hypertrophy with CsA or FK506 [35, 36]. Conversely, previous studies have suggested that CaN inhibitors attenuate hypertrophy in a variety of models [37]. These discrepancies may be explained by the inherent differences between the different models. In addition, the inherent nonspecific effects associated with CsA and FK506 are to be considered. FK506 can also directly alter ryanodine receptor function through its effects on FKBP12 and FKBP12.6, while CsA may influence the leakage of calcium from the sarcoplasmic reticulum through the lipid bilayer [38, 39]. Acute treatment with CsA causes alterations of LTCC activity in disassociated human cardiomyocytes [40]. The current study demonstrated that administration of CsA activated the CaMKII signaling pathway and increased the degree of CH. This is possibly because the specificity of actions by CsA may be associated with their selective interactions with specific CaN subtypes or because of the presence of cell-specific CaN substrates [41].

Three different CnA subtypes are found in mammals: CnA*α* and CnA*β*, which are universally expressed, and CnA*γ* [9]. Furthermore, it has been reported that CnA*β*1 may have a cardioprotective action that decreases inflammation and scar formation [9]. Moreover, mice overexpressing constitutively active CaN show decreased apoptosis after ischemia/reperfusion, whereas deletion of the phosphatase-encoding exon of CnA*β* leads to increased cell death and reduced cardiac function [42, 43]. In contrast, transgenic mice overexpressing an artificially truncated, constitutively active form of CnA*α* lacking the autoinhibitory domain show strong CH and develop HF within the first few weeks of life, which is a response phenocopied by overexpression of a constitutively active form of NFAT [32]. Other studies have suggested that knockout mice lacking the phosphatase domain of CnA*β* show smaller hearts at baseline and exhibit reduced hypertrophy in response to pressure overload, Ang II, or isoproterenol [44]. Notably, in the study by Zhang et al. [36], CsA augmented hypertension but did not prevent CH in spontaneously hypertensive rats. CsA is known to cause numerous unwanted side effects. It has been shown that CsA increases Ang II receptors independently from CaN inhibition, which causes vasoconstriction and systemic hypertension and can promote CH [35, 45–47]. This may be due to CsA alleviating the cardioprotective action of CnA*β*.

There have been some studies on the mechanism of CaMKII and inflammatory pathway regulation [48–53]. Increasing evidence has shown that as a multipurpose kinase, CaMKII plays a pivotal role in many cardiac pathophysiological conditions involving inflammation [54]. Previous studies have shown that TLR4 mediates endothelial inflammation, activation, and dysfunction induced by CaN inhibitors, and according to previous observations, MyD88 silencing in endothelial cells prevented the induction of proinflammatory and endothelial activation markers by CsA and tacrolimus [55]. Our data indicate that, after CsA treatment, the expression of MyD88 and TLR2 obviously increased. The activation of MyD88, NF-*κ*B, TLR2, and TLR4 inflammatory response pathways in pathological states induces an increase in CaMKII protein expression, causes calcium homeostasis, and promotes the occurrence of potentially malignant arrhythmias [56, 57].

WXKL is the first antiarrhythmic Chinese medicine to be approved by the state. Our previous studies indicated that WXKL treatment considerably preserves cardiac function and inhibits arrhythmia by modulating the CaMKII signaling pathway [22, 23]. Moreover, WXKL treats CH and arrhythmia through a mechanism that possibly involves LTCC regulation [20]. We propose a new role for WXKL that may inhibit CH by regulating pathological autophagy [21]. Notably, treatment with WXKL significantly inhibited Ang II-induced hypertrophy in the present study. In addition, WXKL suppressed Ang II-induced elevated expression of CaMKII, CnA, and NFATc4 and prevented Ang II-induced nuclear translocation of NFATc4, suggesting that WXKL attenuated CH by inhibiting the CaMKII and CnA-NFAT signaling pathways. Therefore, owing to its numerous therapeutic benefits, WXKL may be considered as a promising choice for the treatment of CH.

In conclusion, the present study demonstrated that siCaMKII attenuates Ang II-induced CH, which may be partially associated with the downregulation of the CnA-NFAT and MyD88 signaling pathways, and WXKL had a similar effect. Furthermore, siCaMKII may be a promising approach to attenuate the progression of CH and arrhythmia. Our studies define the crosstalk between the CaMKII and CnA-NFAT signaling pathways in vitro, so numerous biological studies are needed for further research in vivo. This will improve the understanding of the mechanisms underlying hypertrophy and may provide evidence for drug application in the treatment of CH.

## Figures and Tables

**Table 1:** CaMKII*δ* (Rat) (NM_012519.2) RNAi site.

	Initiation site	RNAi site sequence
1#	408	TAGAATCTGCCGTCTCTTGAA
2#	754	CCTGGGTATCTTTCTCCAGAA
3#	1141	ACTATGCTGGCTACGAGAAAT
4#	1632	GTACATGGATGGAAATGGAAT

**Table 2:** Inhibition rate of CaMKII*δ* (rat) H9c2-1141 and H9c2-1632 sites after the RNA interference.

Cells	Relative expression	Inhibition rate of CaMKII*δ* (rat)
H9c2-NEG	1	0
H9c2-1141	0.1765726	82.34%
H9c2-1632	0.1890273	81.10%

## Data Availability

The data and materials used in the study are available from the corresponding author upon reasonable request.

## Abbreviations

CaM: Calcium/calmodulin
CaMKII: Calcium-/calmodulin-dependent protein kinase II
CnA-NFAT: Calcineurin A-nuclear factor of activated T cells
CH: Cardiomyocyte hypertrophy
Ang: Angiotensin
siCaMKII: Silenced CaMKII*δ*
CsA: Cyclosporine A
WXKL: Wenxin Keli
I_Ca-L_: L-type Ca^2+^ current
HF: Heart failure
CaN: Calcineurin
HDAC: Histone deacetylase
LTCC: L-type Ca^2+^ channel
RyR2: Ryanodine receptor 2
SR: Sarcoplasmic reticulum
NCX: Na^+^/Ca^2+^-exchanger
BSA: Bovine serum albumin
PBS: Phosphate-buffered saline.

## Supplementary Materials

Supplementary Materials Table S1: list of antibodies. Figure S1: effects of siCaMKII and CsA on the CnA-NAFT signaling cascade; double immunofluorescent staining to observe the effects of siCaMKII, CsA and WXKL on expression of CnA in each group. Figure S2: H9c2-1632: validation of CaMKII*δ* (Rat) 1632 site cell line after RNA interference.
