# Supplementary material for: Cardiac CaMKIIδ and Wenxin Keli Prevents Ang II-Induced Cardiomyocyte Hypertrophy by Modulating CnA-NFATc4 and Inflammatory Signaling Pathways in H9c2 Cells
Source: Evid Based Complement Alternat Med. 2020 Oct 19;2020:9502651. doi: 10.1155/2020/9502651 (PMC7603598; doi:10.1155/2020/9502651)
Supplement: Supplementary Materials — Table S1: list of antibodies. Figure S1: effects of siCaMKII and CsA on the CnA-NAFT signaling cascade; double immunofluorescent staining to observe the effects of siCaMKII, CsA and WXKL on expression of CnA in each group. Figure S2: H9c2-1632: validation of CaMKIIδ (Rat) 1632 site cell line after RNA interference. [file 9502651.f1.docx]

**Supplementary Materials**

**Table S1.** **List of antibodies.**

| Target protein | Dilution | Source |
| --- | --- | --- |
| ANP(FL-153) | 1:200 | Santa Cruz, sc-20158 |
| Anti-BNP antibody | 1:500 | Abcam, ab19645 |
| Anti-CaMKIIδ [ERP13095] antibody | 1:1000 | Abcam, ab181052 |
| Anti-Calcineurin A antibody  Anti-p-CnA (Ser411) | 1:2000  1:1000 | Abcam, ab3673  Pineda Antibody Service |
| Phospho-CaMKII (Thr-286) antibody | 1:1000 | Cell Signaling, 3361S |
| PLB(G-18) | 1:200 | Santa Cruz, sc-20512 |
| Anti-Ryanodine Receptor 2 | 1:1000 | Millipore, AB9080 |
| FKBP 12.6(L-12) | 1:200 | Santa Curz, sc131520 |
| NFATc4(H-74) | 1:200 | Santa Curz, sc13036 |
| GATA-4(C-20) | 1:200 | Santa Curz, sc1237 |
| p-phospholamban (Thr-17)-R | 1:200 | Santa Curz, sc-17024-R |
| Anti-MyD88 antibody | 1:500 | Abcam, ab2064 |
| NF-κB p65(D14E12)XP(R) Rabbit mAb | 1:1000 | Cell Signaling, 8242S |
| Phospho-NF-κBp65(Ser536) (93H1) Rabbit mAb | 1:1000 | Cell Signaling, 3033S |
| Anti-TLR2 antibody [EPNCIR133] | 1:1000 | Abcam, ab108998 |
| TLR4(25) antibody | 1:200 | Santa Curz, sc-293072 |
| p-GATA-4(Ser262) | 1:200 | Santa Curz, sc-1237 |
| p-NFATc4 Antibody (Ser197) | 1:200 | Santa Curz, sc-68708 |

**Fig. S1.**


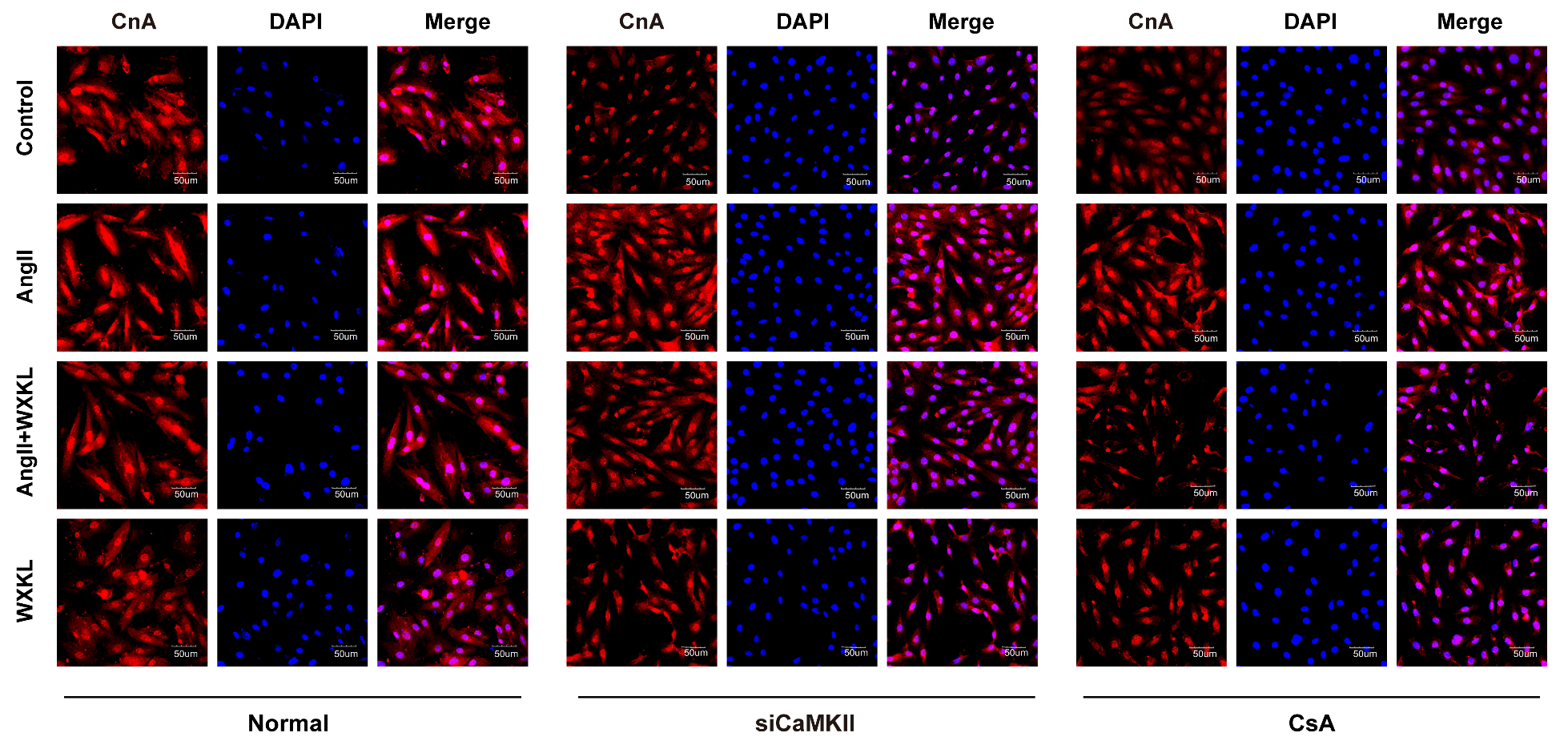


**Fig. S1.** Effects of siCaMKII and CsA on the CnA-NAFT signaling cascade. Double immunofluorescent staining to observe the effects of siCaMKII, CsA and WXKL on expression of CnA in each group. Blue: DAPI staining; Red: CnA; Scale bar: 50 μm.

**Fig. S2.**


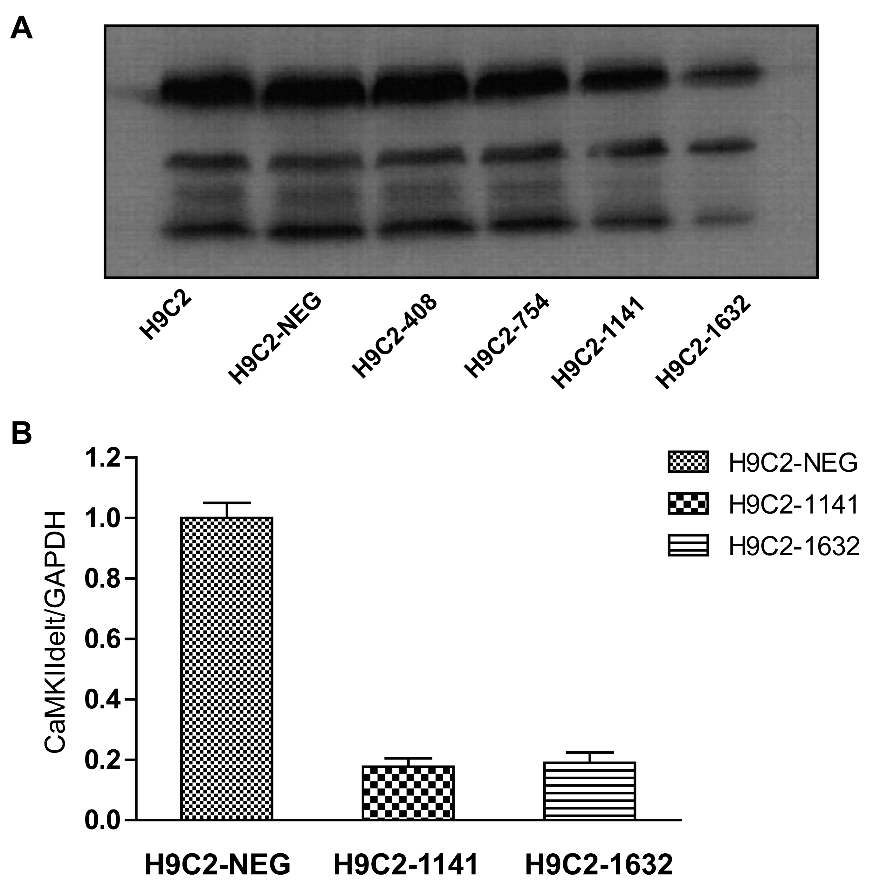


**Fig. S2.** H9C2-1632: Validation of CaMKIIδ (Rat) 1632 site cell line after RNA interference. (A) The expression of CaMKIIδ (Rat) H9C2; H9C2-NEG, H9C2-408, H9C2-754, H9C2-1141 and H9C2-1632 sites after the RNA interference. (B) Relative expression of mRNA of CaMKIIδ (Rat) H9C2-NEG, H9C2-1141, and H9C2-1632 sites after the RNA interference.
